# An Incidental Finding of Libman-Sacks Endocarditis ‎in a Young Female With Systemic Lupus Erythematosus Who Presented With Pleuritic Chest Pain

**DOI:** 10.7759/cureus.49672

**Published:** 2023-11-29

**Authors:** Ali T Alhashem, Walaa M Hassan

**Affiliations:** 1 Internal Medicine, Dammam Medical Complex, Dammam, SAU; 2 Internal Medicine, Alexandria University, Alexandria, EGY

**Keywords:** mitral valve vegetation, anticoagulant therapy, warfarin, systemic lupus erythematous, libman sack endocarditis

## Abstract

Libman-Sacks endocarditis (LSE) is a rare disease found incidentally in ‎postmortem autopsies, characterized by microscopic to large ‎verrucous vegetation on the cardiac valves, the most affected site is ‎the mitral valve followed by the aortic valve. Females of reproductive age ‎were observed as the most affected individuals as found in studies. ‎Most individuals with LSE are asymptomatic and ‎generally discovered lately when they presented with ‎thromboembolic disorders such as stroke, cognitive disabilities, and death. ‎Malignancy and autoimmune diseases involving systemic lupus ‎erythematosus (SLE) and antiphospholipid syndrome (APS) are ‎considered the primary etiology of LSE. As recognized, the majority of LSE cases are ‎asymptomatic, it tends to be challenging to spot the condition ‎at the early pathway of the disease. In this paper, we describe a ‎young female who is known to have SLE on medications, she presented ‎to the emergency department (ED) due to chest pain and exertional ‎dyspnea for a few days, laboratory investigations showed ‎anemia, raised inflammatory marker, and anti-DsDNA. Imaging ‎studies showed bilateral pleural effusion on the chest X-ray ‎and a large vegetation on the posterior mitral valve with moderate regurgitation and normal wall ‎motion in transesophageal echocardiography. The patient was managed by pulse steroid therapy, anticoagulation ‎therapy, and a low dose of diuretic, the patient improved dramatically and ‎discharged home with close follow-up in the clinic. The primary ‎treatment of LSE is anticoagulant therapy, however, surgical ‎intervention should be considered in case of large vegetation ‎recurrent thromboembolism despite anticoagulant therapy. As ‎the prognosis in LSE is considered very poor and there is no definitive laboratory investigation ‎exists to confirm the diagnosis, we highlight the importance of ‎considering LSE as a serious and crucial differential diagnosis ‎when dealing with SLE patients who presented with dyspnea and pleural effusion secondary to valvular dysfunction, mainly the mitral valve.

## Introduction

Systemic lupus erythematosus (SLE), one of the most prevalent ‎chronic relapsing inflammatory autoimmune diseases, affects several ‎organ systems and manifests clinically in a variety of ways [1]. ‎According to estimations, there are 3.41 million patients with SLE ‎worldwide, with a prevalence of 43.7 (15.87 to 108.92) per 100,000 ‎people. United Arab Emirates had the highest estimated incidence of ‎SLE (166.92, 139.01 to 198.54 per 100 000 people) in the world [2]. ‎Most studies found that the incidence of SLE in females peaks in the middle and younger age, whereas the peak incidence for men often occurs later, between ‎the fifth and seventh decades [3]. Despite the enhanced chance of ‎survival, SLE patients' mortality rates are estimated to be 1.4-5 times ‎higher than those of the general population [4]. As was observed in ‎many studies, the survival rates at 5, 10, and 15 years after the ‎diagnosis were 96%, 93%, and 76%, respectively [5].

SLE individuals may have a variety of systemic symptoms, ranging ‎from general symptoms, such as fever, malaise, myalgia, and weight ‎loss, to specific systemic manifestations involving cutaneous, ‎musculoskeletal, renal, and cardiovascular organ systems [6]. ‎Cardiovascular involvement in SLE results from a synergistic ‎combination of pathogenic processes, resulting in the development ‎of many cardiac events at a younger age than the general ‎population. The most prevalent cardiac outcomes in SLE patients are ‎pericarditis, myocarditis, valve disorders, and conducting system ‎disorders [7].‎

According to estimations, one in every 10 SLE patients had Libman-Sacks endocarditis (LSE). LSE is a sterile lesion that occurs ‎concurrently with SLE and antiphospholipid syndrome (APS) ‎targeting the mitral and aortic valve, causing stenosis or regurgitation ‎‎[7]. A hypercoagulable condition is commonly linked with ‎nonbacterial thrombotic endocarditis such as in solid tumors, SLE, and APS [8]. LSE patients are usually asymptomatic, however, they ‎might have a variety of presenting symptoms, in terms of arrhythmia, ‎pericarditis, and heart failure secondary to mitral valve regurgitation, ‎but according to data, valve vegetation increases the risk of ‎embolic cerebrovascular disease, which they might present with ‎ischemic stroke or transient ischemic attack (TIA) [8,9].‎

We reported a young female who had SLE presented to ‎ED because of chest pain and shortness of breath and was incidentally found ‎to have a large thrombus versus vegetation on the posterior mitral valve through ‎transesophageal echocardiography, Finally, the patient was labeled to ‎have LES and started on anticoagulant therapy. The aim of the report ‎is to expand the knowledge, awareness, and importance of early ‎detection of LSE in SLE patients to prevent ‎cerebrovascular and cardiovascular complications and disabilities.‎

## Case presentation

We report a 17-year-old Saudi female, a single, student, known to have ‎systemic lupus erythematosus disease (SLE). In 2020, her initial ‎presentation was a butterfly rash on her face, arthralgia in the small ‎joints of her hands, and hair loss. ‎The antinuclear antibody (ANA) and anti-DsDNA were positive. The SLE diagnosis was ‎confirmed and prednisolone and hydroxychloroquine were initiated as a ‎therapy.‎

Two years apart from the disease diagnosis and remission state, in ‎late August of 2022, the patient started to have gradual left-side chest ‎pain for four days with strict compliance to her home medications, the pain is not radiating or referring to any site of the body, ‎associated with exertional dyspnea and dry cough. After four days, ‎the patient came to the emergency department with that complaint and ‎was examined. Up on examination, the patient was conscious, alert, ‎oriented, and not in respiratory distress, but in mild-moderate pain. ‎Vitally, she was afebrile at 37.4c, heart rate 110 bpm, respiratory rate ‎‎20/minute, blood pressure 135/65 mmHg, and oxygen saturation (96% on ‎room air). On oral cavity exam, good hygiene, and no ulcer. During the neck ‎exam, no swelling or lymphadenopathy was appreciated. Chest ‎auscultation reduced air entry bilaterally in the base of the lungs ‎and other chest exams were unremarkable. On cardiovascular examination, ‎jugular venous pressure (JVP) was elevated at about 11 cm. On auscultation, the patient had normal first and second ‎heart sounds with no added sounds, and no parasternal heave was ‎appreciated. Abdominal examination revealed a soft, non-tender abdomen, and no ‎hepatosplenomegaly. In lower limb examination, patent peripheral pulses, ‎grade +1 edema in both legs, no erythema, and negative ‎signs for deep vein thrombosis.‎

Laboratory and imaging studies

On arrival at the emergency department (ED), blood samples were ‎taken, which showed low hemoglobin 7.1 g/dL, total white cells 5.5x10-9/L, lymphocyte ‎count 0.36x10-9/L, high anti-DsDNA antibody, high ESR 137 mm/hr, CRP was ‎‎11.8 mg/dL, and normal urea/creatinine level. C3 and C4 were low. Septic work was sent for three sets of blood cultures, and it became negative for ‎organisms. The electrocardiograph showed a normal sinus rhythm ‎with low voltage on limb leads.‎

**Figure 1:**
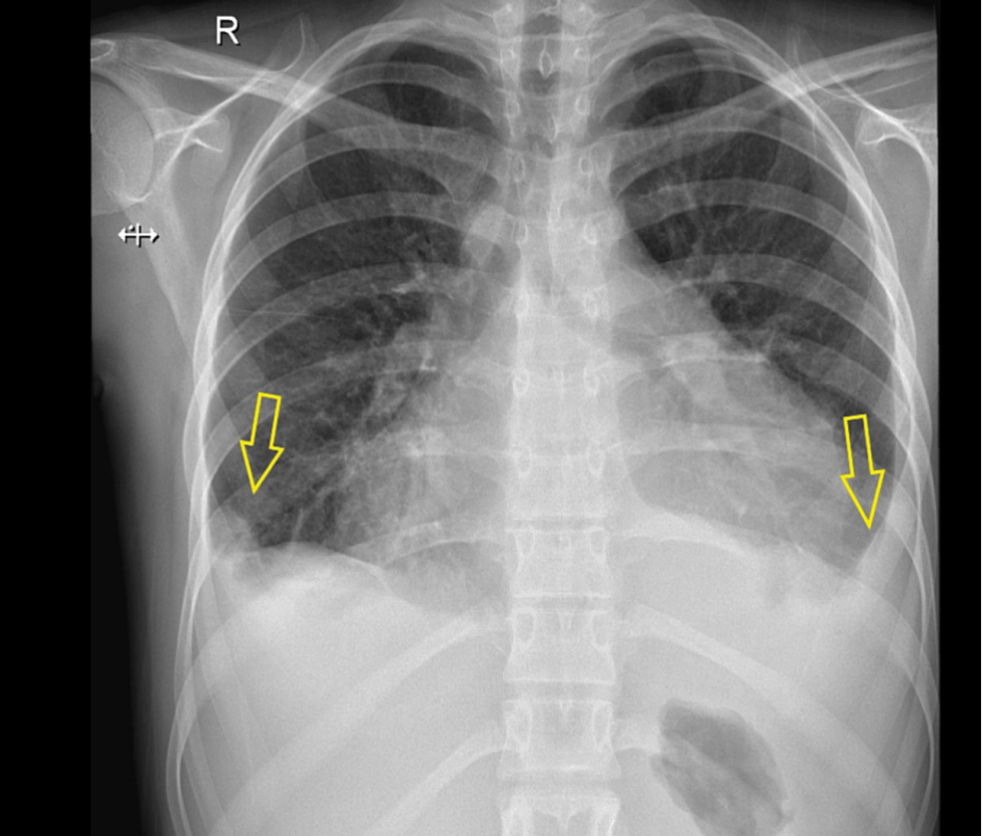
Initial chest imaging before initiating the management plan Image description: posterior-anterior view of the erect chest X-ray showing cardiomegaly with a silhouette sign and bilateral obliteration of costophrenic angle more in the left side, which goes with bilateral pleural effusion.

**Figure 2:**
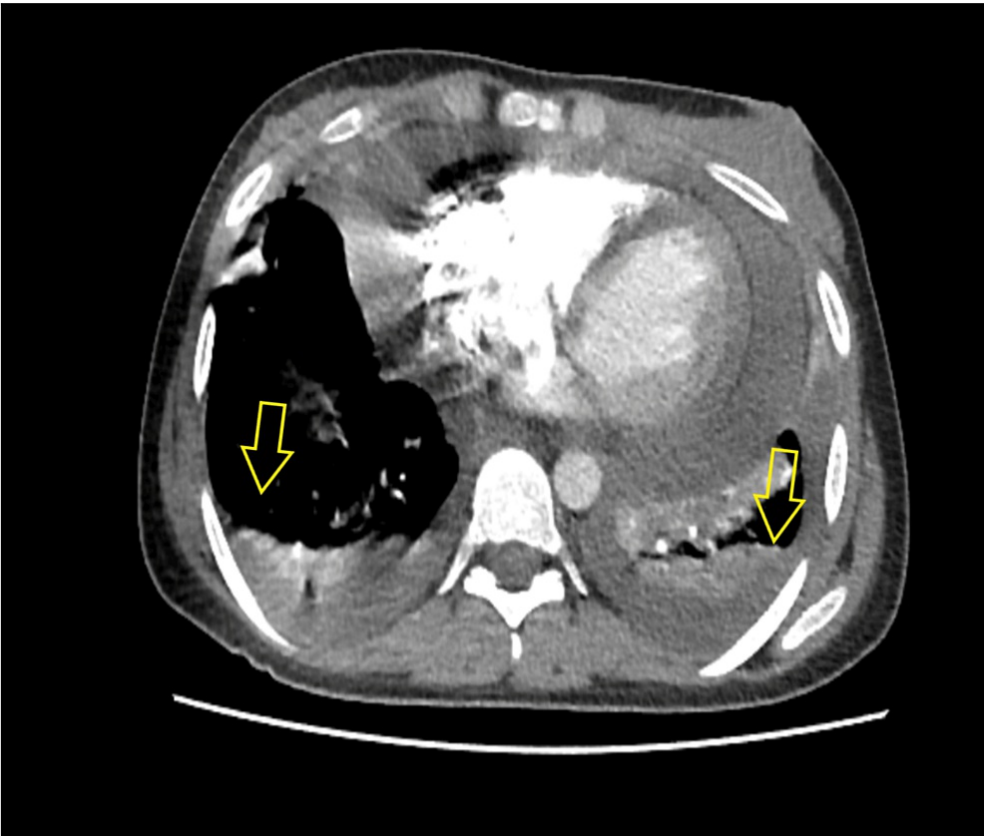
A cross-sectional computer scan study with contrast showing pericardial effusion with passive lung atelectasis more on the left side Lung parenchyma shows a diffuse bilateral mosaic attenuation pattern.

The chest X-ray showed bilateral pleural effusion with a flask cardiac ‎silhouette (Figure 1). A computerized tomography (CT) scan conducted and ‎was showed bilateral pleural effusion with pericardial effusion (Figure 2).

**Figure 3:**
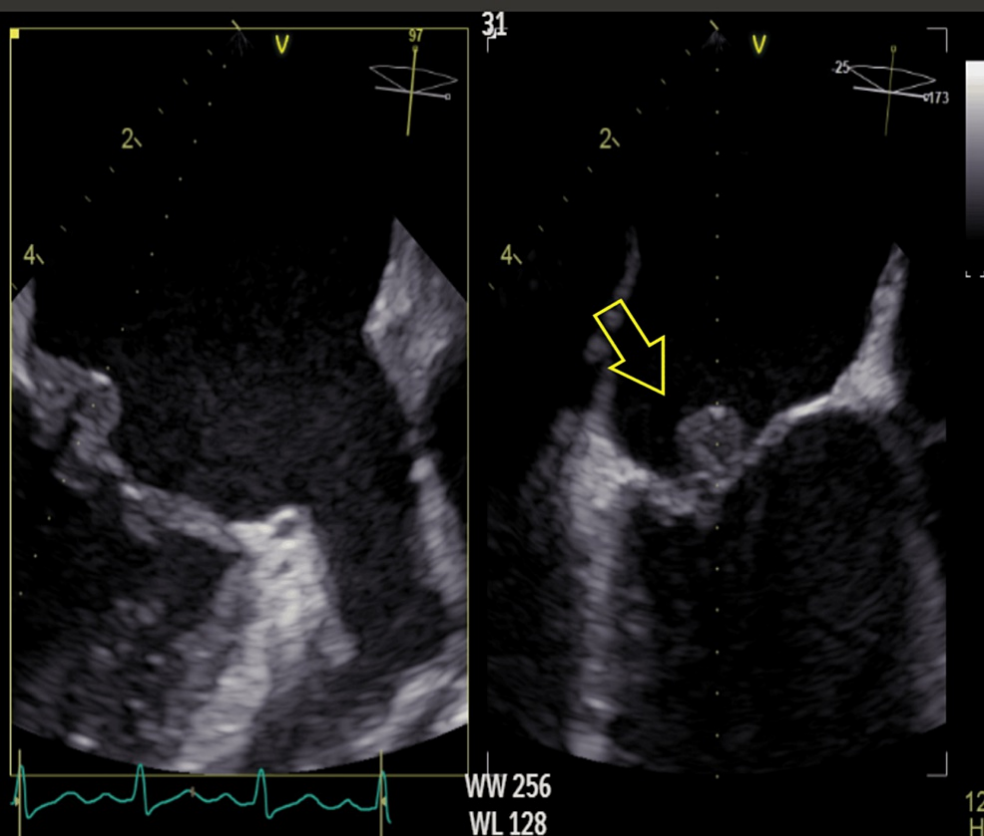
Transesophageal echocardiography shows a bulky thrombus/mass on the posterior mitral valve leaflet measuring 8 x 18 mm

**Figure 4:**
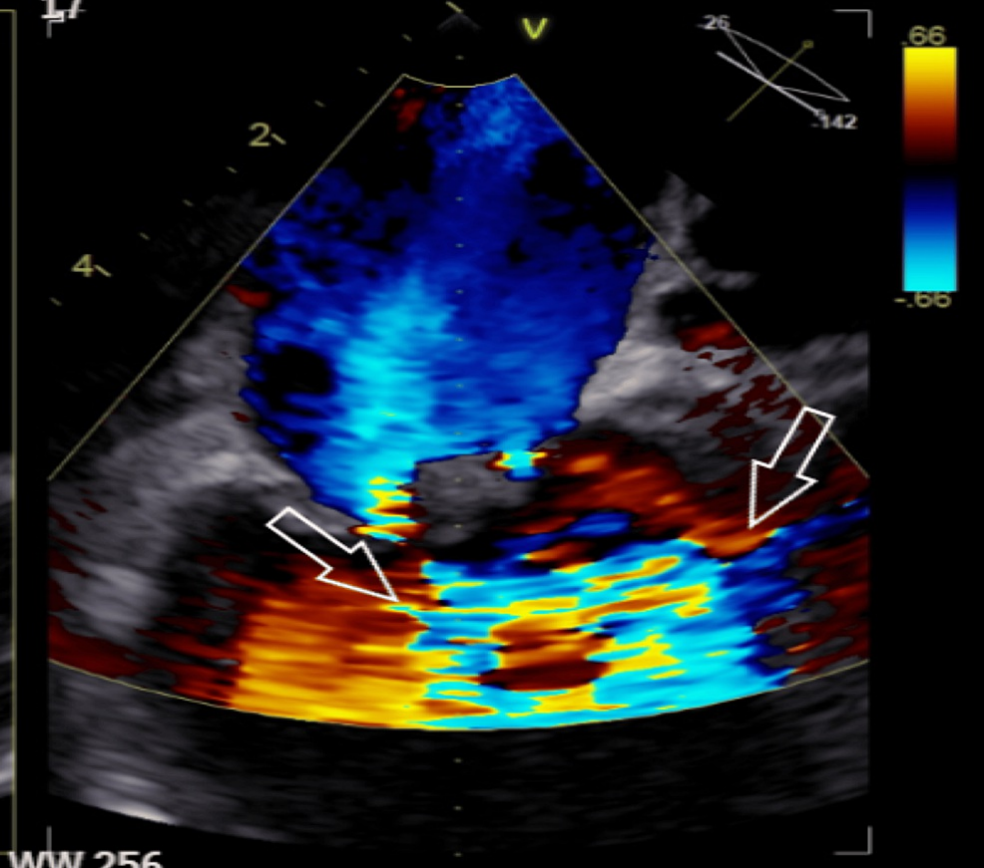
Transesophageal echocardiography shows moderate mitral regurgitation

‎Transesophageal echocardiography (TEE) was requested urgently, ‎and it showed a bulky mass/thrombus on the posterior mitral ‎valve about 8 x 18 mm, the left ‎ventricle was normal in size and systolic function, the right ventricle ‎was normal in size and function as well (Figures 3-4). The TEE findings were highly ‎suggestive of LSE. An abdominal ultrasound was requested and showed ‎minimal ascitic fluid‎.

**Figure 5:**
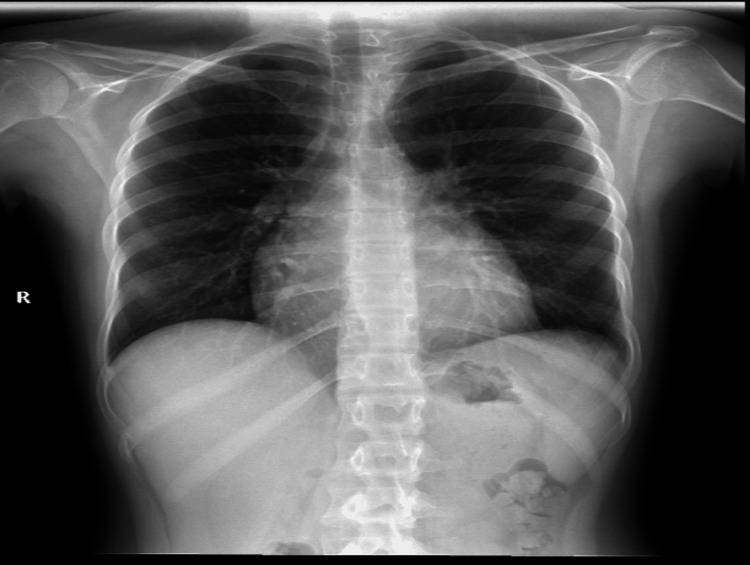
Chest X-ray repeated after five days of pulse steroid therapy and a low dose of diuretics, which shows a huge improvement in comparison to the previous one, in terms of improvement in the pleural effusion and cardiomegaly

The patient was admitted to the regular ward with an SLE flare in terms of serositis ‎and LSE for pulse steroid therapy and therapeutic anticoagulation. ‎The patient was started on methylprednisolone as a pulse therapy, bridging ‎the anticoagulation information of low molecular weight heparin to warfarin ‎and a low dose of furosemide 40 milligrams intravenous once daily for ‎‎three days. Five days later, the patient showed a significant improvement ‎after receiving pulse steroid therapy and therapeutic anticoagulation ‎with no complications (Figure 5). The patient was discharged home safely on a tapering dose of prednisolone (2 mg/kg) and warfarin with close ‎outpatient follow-up within three days for INR monitoring and within two weeks for SLE flare.‎

## Discussion

A variety of cardiac and vascular complications can develop in SLE ‎‎patients, affecting the pericardium, myocardium, valves, coronary ‎‎arteries, and conduction system. LSE is an uncommon disorder with ‎‎prevalence in an autopsy series, ranging from 0.9% to 1.6% that is ‎‎most frequently discovered post-mortem [10,11]. In the ‎‎demonstrated case, the primary etiology for the verrucous vegetation ‎‎was SLE; interestingly, many studies of autoptic cases who are found to have LSE are more often ‎‎diagnosed with concomitant cancer, which reflects that SLE is not only the primary etiology of LSE, cancer as well can cause verrucous vegetation in non-SLE patients. On the other hand, patients ‎‎with a history of previous cardiac and valvular surgery were more ‎‎likely to have IE, which increases the risk of bacterial or fungal vegetation [12].‎

The majority of SLE patients who have LSE are asymptomatic; ‎‎however, LSE individuals are more likely to present with ‎‎complications of LSE such as cerebrovascular embolism (stroke or ‎‎transient ischemic attack) and systemic embolism (mesenteric, limb ‎‎ischemia, or peripheral arterial embolism) [8]. As described in the ‎‎previous studies, many individuals who have LSE are either ‎‎asymptomatic or present with thromboembolism manifestations. ‎However, our patient presented with heart failure manifestations ‎‎with preserved ejection fraction secondary to SLE flare in terms of serositis and moderate mitral valve regurgitation with verrucous vegetation. There are few ‎‎studies in Saudi Arabia that were documented and published for SLE ‎‎patients who presented with heart failure symptoms secondary to an SLE flare in terms of serositis and LSE with preserved ejection fraction.‎

The diagnosis of LSE in SLE patients is challenging, yet there is no ‎‎definitive laboratory test to confirm the diagnosis of LSE. A high clinical suspicion of LES will lead to the diagnosis, especially in SLE patients who have pleural effusion either secondary to SLE flare in terms of serositis or mitral valve regurgitation secondary to LSE, a full assessment should ‎be ‎done with laboratory studies. The most important two investigations in LSE are three blood cultures ‎before ‎starting empirical antibiotics and transoesophageal ‎‎echocardiography. A verrucose vegetation in LSE has ‎‎specific characteristics, mainly irregular borders, absence of ‎‎independent motions, heterogeneous echo density, and involving ‎the ‎basal and mid-portion of the mitral and aortic valves [8].‎

The core management of LSE is systemic anticoagulation regardless ‎‎of the underlying cause (malignancy or SLE). Low molecular weight ‎‎heparin (LMWH) or unfractionated heparin at therapeutic doses ‎‎should be utilized instead of factor Xa inhibitors (such as apixaban, ‎‎rivaroxaban, and dabigatran) if APS has not been ruled out. On the ‎‎other hand, in the setting of autoimmune or inflammatory disorders, ‎‎warfarin can be used with an extended survival rate in LSE [13]. All ‎‎individuals who receive anticoagulant therapy, especially on ‎warfarin ‎must be educated in a written order for drug interaction ‎and the ‎need for regular follow-up in an anticoagulant clinic. In our ‎‎presenting case, the patient presented with SLE flare-up in terms of ‎‎serositis with pleural effusion, chest pain, and tachycardia with low ‎‎complement serology, started on pulse ‎‎methylprednisolone therapy for three days, therapeutic LMWH and ‎‎furosemide for pleural effusion, the patient showed a huge ‎‎improvement and was discharged home safely on mycophenolate, ‎‎hydroxychloroquine, prednisolone, and warfarin with close follow-up. Pulse steroid therapy should be considered in severe SLE flares ‎‎‎(e.g., central nervous system, renal system) as induction therapy for ‎‎rapidly controlling the flares and reducing inflammatory process, ‎‎however, minimizing exposure must always be made to prevent long-term complications (opportunistic infection, gastric ulcer, and ‎‎osteoporosis) [14].‎

As reviewed in many studies, valvular vegetation and ‎‎regurgitation are seen in up to 61% of SLE patients. Surgical ‎‎intervention in LSE has a crucial role and a strong indication in case of ‎‎giant vegetation and recurrent embolization despite therapeutic ‎‎anticoagulation. A mechanical valve is preferred among young ‎patients ‎with APS who require lifelong anticoagulants. The risks and ‎benefits ‎should be weighed while considering surgery in relation to ‎the ‎underlying cause and life expectancy [15].‎

## Conclusions

LSE is a fatal clinical condition if it is not discovered early. As ‎most of the individuals are asymptomatic, they mainly ‎present with complications of LSE such as stroke, mesenteric ‎ischemia, or limb ischemia. Since there are no laboratory tools to ‎make a definitive diagnosis of LSE, high clinical judgment and ‎suspicion need to be considered in individuals with SLE who ‎presented with either pleural effusion secondary to mitral valve regurgitation and vegetation or in late complications such as thromboembolic events. Transesophageal ‎echocardiography has a crucial role in diagnosing LSE. The ‎core treatment of LSE is anticoagulant therapy. However, surgical ‎intervention can be considered in case of recurrent thromboembolic ‎events and in the presence of large vegetation. The prognosis is poor, ‎as LSE individuals are prone to recurrent ‎thromboembolic ‎events, cognitive disability, and death. The aim of this case report is ‎to expand the knowledge and awareness of early detection and ‎management of LSE in terms of preventing further complications and ‎disabilities.‎

## References

[1] Mohammadi Kebar Y, Avesta L, Habibzadeh A, Hemmati M (2019). Libman-Sacks endocarditis in patients with systemic lupus erythematosus with secondary antiphospholipid syndrome. Caspian J Intern Med.

[2] Tian J, Zhang D, Yao X, Huang Y, Lu Q (2023). Global epidemiology of systemic lupus erythematosus: a comprehensive systematic analysis and modelling study. Ann Rheum Dis.

[3] Rees F, Doherty M, Grainge MJ, Lanyon P, Zhang W (2017). The worldwide incidence and prevalence of systemic lupus erythematosus: a systematic review of epidemiological studies. Rheumatology (Oxford).

[4] Bultink IE, de Vries F, van Vollenhoven RF, Lalmohamed A (2021). Mortality, causes of death and influence of medication use in patients with systemic lupus erythematosus vs matched controls. Rheumatology (Oxford).

[5] Doria A, Iaccarino L, Ghirardello A (2006). Long-term prognosis and causes of death in systemic lupus erythematosus. Am J Med.

[6] Cojocaru M, Cojocaru IM, Silosi I, Vrabie CD (2011). Manifestations of systemic lupus erythematosus. Maedica (Bucur).

[7] Alghareeb R, Hussain A, Maheshwari MV, Khalid N, Patel PD (2022). Cardiovascular complications in systemic lupus erythematosus. Cureus.

[8] Ibrahim AM, Siddique MS (2023). Libman-Sacks Endocarditis. https://www.ncbi.nlm.nih.gov/books/NBK532864/.

[9] Mary C Rodriguez Ziccardi (2020). Libman-Sacks endocarditis. MedScape. Medscape.

[10] Eiken PW, Edwards WD, Tazelaar HD, McBane RD, Zehr KJ (2001). Surgical pathology of nonbacterial thrombotic endocarditis in 30 patients, 1985-2000. Mayo Clin Proc.

[11] Llenas-García J, Guerra-Vales JM, Montes-Moreno S (2007). Nonbacterial thrombotic endocarditis: clinicopathologic study of a necropsy series. Rev Esp Cardiol.

[12] Bussani R, DE-Giorgio F, Pesel G (2019). Overview and comparison of infectious endocarditis and non-infectious endocarditis: a review of 814 autoptic cases. In Vivo.

[13] Bauer KA (2022). Nonbacterial thrombotic endocarditis. UpToDate.

[14] Wallance DJ (2023). Overview of the management and prognosis of systemic lupus erythematosus in adults. UpToDate.

[15] Abouelela Y, Gukop P, Livesey S (2021). Surgical management of Libman-Sacks mitral valve endocarditis. Int J Surg Case Rep.

